# Mitochondrial Dysfunction, Oxidative Stress, and Therapeutic Strategies in Diabetes, Obesity, and Cardiovascular Disease

**DOI:** 10.3390/antiox12030658

**Published:** 2023-03-07

**Authors:** Karina-Alexandra Cojocaru, Ionut Luchian, Ancuta Goriuc, Lucian-Mihai Antoci, Cristian-Gabriel Ciobanu, Roxana Popescu, Cristiana-Elena Vlad, Mihaela Blaj, Liliana Georgeta Foia

**Affiliations:** 1 Department of Biochemistry, Faculty of Dental Medicine, “Grigore T. Popa” University of Medicine and Pharmacy, 16 Universității Street, 700115 Iasi, Romania; 2 Department of Periodontology, Faculty of Dental Medicine, “Grigore T. Popa” University of Medicine and Pharmacy, 16 Universității Street, 700115 Iași, Romania; 3 Department of Medical Genetics, Faculty of Medicine, “Grigore T. Popa” University of Medicine and Pharmacy, 16 Universității Street, 700115 Iasi, Romania; 4 Department of Medical Genetics, “Saint Mary” Emergency Children’s Hospital, Vasile Lupu Street, No. 62, 700309 Iasi, Romania; 5 Department of Internal Medicine, Faculty of Medicine, “Grigore T. Popa” University of Medicine and Pharmacy, 16 Universității Street, 700115 Iasi, Romania; 6 Department of Nephrology-Internal Medicine, “Dr. C. I. Parhon” Clinical Hospital, 700503 Iasi, Romania; 7 Anaesthesia and Intensive Care Department, Grigore T. Popa University of Medicine and Pharmacy, 700115 Iasi, Romania; 8 Anaesthesia and Intensive Care Department, Sf. Spiridon University Hospital, 700111 Iasi, Romania

**Keywords:** mitochondria, ROS, diabetes, obesity, cardiovascular disease, antioxidants

## Abstract

Mitochondria are subcellular organelles involved in essential cellular functions, including cytosolic calcium regulation, cell apoptosis, and reactive oxygen species production. They are the site of important biochemical pathways, including the tricarboxylic acid cycle, parts of the ureagenesis cycle, or haem synthesis. Mitochondria are responsible for the majority of cellular ATP production through OXPHOS. Mitochondrial dysfunction has been associated with metabolic pathologies such as diabetes, obesity, hypertension, neurodegenerative diseases, cellular aging, and cancer. In this article, we describe the pathophysiological changes in, and mitochondrial role of, metabolic disorders (diabetes, obesity, and cardiovascular disease) and their correlation with oxidative stress. We highlight the genetic changes identified at the mtDNA level. Additionally, we selected several representative biomarkers involved in oxidative stress and summarize the progress of therapeutic strategies.

## 1. Introduction

Mitochondria have a unique double-membrane structure and represent the central site of metabolism, displaying morphologies, dynamics, and functions specific to each cell and tissue [1]. They are responsible for the bulk cellular adenosine triphosphate (ATP) production, through the process of oxidative phosphorylation (OXPHOS). Mitochondria are also the site of important biochemical pathways, including the tricarboxylic acid cycle and a part of the ureagenesis cycle or haem synthesis. They are an important regulator of cell apoptosis and cytoplasmic calcium concentration [2,3,4]. Mitochondrial deoxyribonucleic acid (mtDNA) is essential for proper mitochondrial function [2]. Furthermore, mitochondria are also one of the most important intracellular sites for the formation of reactive oxygen species (ROS). ROS are a family of free radicals that includes superoxide anions, hydroxyl and peroxyl radicals, as well as other compounds capable of generating free radicals [5]. Cells possess numerous systems to counteract the effect of ROS, but their excessive production has been associated with damage at the protein, lipid, and DNA levels [5,6].

Pathophysiological changes at the mitochondrial level have recently been associated with metabolic pathologies, neurodegenerative diseases, cellular aging, and cancer. The pathological mechanisms underlying these changes are still unknown. In this article, we describe the genetic changes identified at the mitochondrial level in metabolic pathologies and their connection with oxidative stress. Metabolic syndrome (MS) represents the accumulation of signs and conditions that, altogether, increase the risk of developing cardiovascular disease, stroke, and type II diabetes mellitus (T2DM). MS is present if at least three of the following five criteria are met: abdominal obesity, high blood pressure, high blood sugar, high serum triglycerides, and low serum high-density lipoprotein [7,8,9]. It represents a major health issue in today’s society and is significantly connected to socioeconomic difficulties encountered around the world. Early identification, diagnosis, and treatment can improve the long-term life quality and health status of patients with metabolic syndrome [10]. The selected and described pathologies (cardiac, obesity, and diabetes) are directly related to oxidative changes generated by excessive ROS and mitochondrial dysfunctions. Therefore, to understand the involvement of mitochondria in these pathologies, we must describe the structure and function of mitochondria, the physiological mechanisms of its biogenesis, and ROS generation at this level.

## 2. Mitochondrion Structure and Function

### 2.1. The Electron Transport Chain

**Figure 1:**
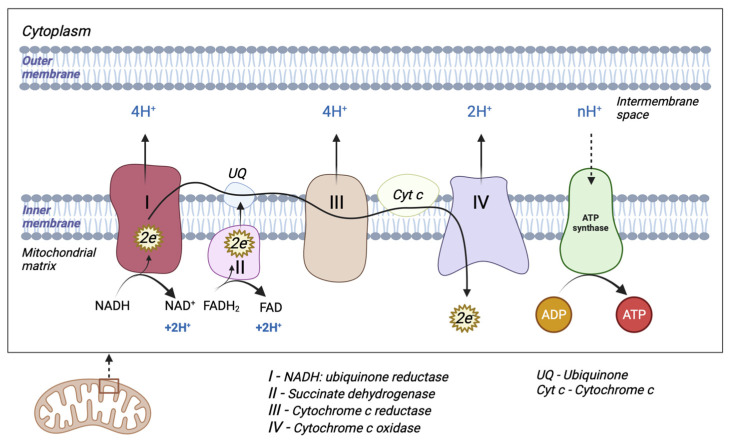
Schematic representation of mitochondrial electron transport chain (ETC). The ETC consists of five enzyme complexes (I, II, III, IV, and V).

The mitochondrion is a cytoplasmic organelle that consists of a double membrane, matrix, and mtDNA. The outer membrane and intermembrane space are relatively permeable in contrast to the inner membrane which has a restrictive permeability, containing the enzymes required for electron transport [11]. Mitochondria generate the majority of cellular energy in the form of ATP, through the oxidation of reduced nicotinamide adenine dinucleotide (NADH) and reduced flavin adenine dinucleotide (FADH2), and subsequently, the process of oxidative phosphorylation [12]. Molecules derived from the catabolism of glucose (glycolysis), fatty acids (beta-oxidation), and amino acids (deamination/transamination) are further referred to the tricarboxylic acid (TCA) cycle to generate the required OXPHOS substrate [13]. An electrochemical gradient generated at the level of the inner membrane generates OXPHOS. The electron transport chain (ETC) is made up of five enzyme complexes (I, II, III, IV, and V), located at the level of the mitochondrial inner membrane. Electrons donated by NADH/FADH2 coenzymes are transferred to complex I (NADH: ubiquinone reductase) or complex II (succinate dehydrogenase) of the ETC [14]. The two electrons from NADH are given to ubiquinone (UQ) with the help of cofactors. Subsequently, ubiquinone is reduced to ubiquinol (UQH2). This transfer of electrons triggers the introduction of protons from the matrix into the intermembrane space (through the transfer of two electrons, four protons are introduced). Electrons donated by FADH2 are transferred to the UQ via complex II but are not associated with the transport of protons from the matrix into the intermembrane space [13]. Afterward, they are transferred to complex III (cytochrome c reductase), made up of cytochromes b and c1. The entire process of electron transfer from UQH2 to cytochrome c is called the Q cycle. Initially, UQH2 binds to complex III, facilitating the access of two protons in the intermembrane space, while two electrons are released, following different paths. The first electron is transferred to cytochrome c1 (at this level it reduces Fe^3+^ to Fe^2+^), and from this level, it is then transferred to cytochrome c. The second electron is given to cytochrome b; subsequently, UQ is partially reduced to a molecule called the semi-quinone radical ion (Q-). In the second stage, a new UQH2 molecule is attached to complex III following the same pattern; therefore, a new electron is bound to the cytochrome c level, and the second electron is bound at the Q level with the formation of a UQH2 molecule. At the end of this process, four protons are generated in the intermembrane space. Four electrons are transferred from four cytochrome c molecules to complex IV (cytochrome c oxidase), where molecular oxygen is bound and reduced to water. Finally, at the level of complex IV, eight protons are transferred from the matrix (four are used for the formation of two water molecules, and the other four are transferred to the intermembrane space) [15]. At the end of the electron transport process, using one molecule of NADH, 10 protons are generated towards the intermembrane space (two from complex IV and four each from complex I and complex III, respectively). In this way, an electrochemical gradient known as mitochondrial membrane potential is produced. Complex V (F0F1 ATP synthase) consists of two domains: extramembrane (F1) and transmembrane (F0). This transport of electrons is associated with the transport of protons from the level of the internal membrane, generating the electrochemical gradient that is necessary for ATP production [13,14,16] (Figure 1).

### 2.2. Mitochondrial DNA Structure

mtDNA nucleotide sequences were first identified in 1981, and were further re-evaluated and subsequently revised in 1999 [17,18]. mtDNA is a double-stranded circular DNA molecule consisting of 16,569 bp which encodes 37 genes, including 13 polypeptides essential for the OXPHOS mechanism, 2 ribosomal RNAs (12S and 16S), and 22 transfer RNAs. mtDNA has a special structure compared to genomic DNA; it does not contain introns, as genes have absent or reduced portions of non-coding bases between them [19].

Zong et al. described free circulating mtDNA in blood samples with an important prognostic role in various cancers, cardiac arrest, and sepsis [20]. Subsequently, circulating free mtDNA was identified as a major mediator of innate immunity and systemic inflammatory response. The process of being released into plasma (by an unknown mechanism) results in the activation of neutrophils, mediated by the Toll-like receptor 9 (TLR9) [21]. mtDNA is also found in the cytosol. It has been shown that oxidative stress, viral or bacterial infections, or miss-packaging lead to its release and are involved in innate intracellular immune responses [22]. Mitochondrial dysfunctions have been correlated with obesity, diabetes mellitus, and cardiovascular pathologies. An increased amount of glucose is predisposed to the increased production of ROS, with destructive effects at the mitochondrial level [23]. The aging process, the reduced action capacity of antioxidants, and the changes produced at the mitochondrial level can be as important causes of metabolic pathologies.

### 2.3. Mitochondrial Biogenesis and Dynamics

Most mitochondrial proteins are nuclear-encoded proteins and are translated by cytosolic ribosomes, processed, and imported into the mitochondria via the TIM/TOM system [24]. The TOM complex is the translocase of the outer mitochondrial membrane and mediates the importing of nuclear-encoded proteins into the intermembrane space [25]. There are two distinct mitochondrial translocase complexes in the inner mitochondrial membrane (TIM) [26]. The TIM22 and TIM23 complexes recognize and import different classes of proteins [27]. Mitochondrial dynamics is essential in maintaining mitochondrial homeostasis and is achieved through two processes: fusion and fission. Imbalances between the two events generate mitochondrial morphological changes, an excess of fission causes the formation of fragmented mitochondria, and an excess of fusion triggers mitochondria elongation.

Mitofusins (Mfn) 1 and 2 are proteins involved in the fusion process of the outer mitochondrial membrane. The fusion of the outer mitochondrial membrane is most often achieved simultaneously with the fusion of the inner membrane, with the latter being mediated by the optic atrophy 1 protein (OPA1). The absence of Mfn cuts off the fusion phenomenon of both membranes. Mitochondrial fission is regulated by dynamin-related protein 1 (Drp1) and fission protein (Fis1) [28]. Under various metabolic conditions, several disbalances in such proteins occur during hyperglycaemic conditions, and Drp1 and Fis1 are increased, while Mfn1, Mfn2, and OPA1 are reduced [29].

Mitochondrial biogenesis is a complex process through which cells increase their mitochondrial mass and require coordination between nuclear and mitochondrial DNA. This process involves mtDNA transcription and translation processes, and the synthesis, import, and association of mitochondrial proteins encoded by nuclear DNA [30].

Mitochondrial biogenesis dysfunction has been associated with metabolic disorders such as obesity and T2DM. A decline in the proliferator-activated receptor gamma coactivator-1α (PGC-1α), AMP-activated protein kinase (AMPK), and silent information regulator 1 (SIRT-1) signalling pathways seems to be the underlying mechanism for reduced mitochondrial biogenesis in the diabetic kidney and the diabetic heart as well, with hypoadiponectinemia being reported to impair AMPK-PGC-1α signalling [30].

### 2.4. Mitophagy

Autophagy is a natural mechanism which was highly conservated throughout evolution, by which the useless cytoplasmic material is transported to lysosomes for destruction [31]. Autophagy is influenced by a variety of factors. The autophagic response promotes the adaptation to stress and increases cellular viability [32]. Components of the autophagy response are implicated in regulated cell death [33].

The degradation of mitochondria through selective autophagy is referred to as mitophagy, a process that involves the selective sequestration of damaged or dysfunctional mitochondria into double-membraned autophagosomes for later lysosomal destruction. Mitophagy has been described in mammalian cells as being facilitated by two well-studied pathways, ubiquitin-mediated and receptor-mediated, and is essential for maintaining cellular fitness [34,35].

Mitophagy ubiquitin-mediated pathways are regulated by two key proteins PTEN-induced putative kinase protein 1 (PINK1) and Parkin. Normally, PINK1 is imported into healthy mitochondria via the TIM/TOM system and further degraded by proteolytic reactions. Damaged mitochondria lose membrane potential, which impairs the TIM/TOM system’s function, resulting in the accumulation of PINK1 on the outer mitochondrial membrane, which promotes the recruitment of Parkin and the activation of its ubiquitination ligase activity, leading to the ubiquitination of proteins from the outer mitochondrial membrane. Further, Parkin promotes the recruitment of autophagy adaptors, such as optineurin (OPTN) and nuclear dot protein 52 kDa (NDP52), leading to the degradation of damaged mitochondria [36,37,38].

The mitophagy receptor pathway is mediated by receptors embedded in the outer mitochondrial membrane, most notably by NIX (known as BCL2 interacting protein 3 like (BNIP3L)), BCL2 interacting protein 3 (BNIP3), and FUN14 domain containing 1 (FUNDC1), which are characterized by the presence of an LC3-interacting region (LIR) that can directly bind to the autophagy mediator LC3 to promote mitophagy when mitochondria are damaged [34,39].

Mitophagy is implicated in insulin resistance and some cardiac pathological conditions. The dysfunctional mitophagy mechanism has been linked to the development of insulin resistance [40]. Moreover, an efficient mitophagical response helps the cardiomyocytes to survive during the nutritional stress in myocardial infarction [41].

## 3. Oxidative Stress and Mitochondrial Dysfunctions

Mitochondrial dysfunctions generated by ROS production in the OXPHOS process are caused by mitochondrial and cellular component damage (DNA, lipids, proteins, and other molecules) [42]. Metabolic disorders involve the coexistence of numerous risk factors, such as obesity, abnormal cholesterol, and triglyceride values or blood pressure [8].

Oxidative stress is characterized by the imbalance between the production of ROS and the action of antioxidants, with the destructive effect of ROS. Most ROS are generated at the complex I and III levels of the mitochondrial respiratory chain by releasing electrons from NADH and FADH2 to the ETC [43]. Free radicals have an increased reactivity due to unpaired electrons, with ROS being one of the most significant recognized classes [44,45,46]. At the mitochondrial level, through the acceptance of an electron by the molecular oxygen, the superoxide anion (O_2_^•−^) is generated. It interacts with other molecules or generates secondary ROS [46]. O_2_^•−^ is subsequently transformed into a more stable compound, hydrogen peroxide (H_2_O_2_), under the action of the enzyme superoxide dismutase (SOD). The considerable presence of this enzyme at the mitochondrial level supports the importance of O_2_^•−^ elimination [23,45,47,48]. H_2_O_2_ can be transported through aquaporins, present at the level of the inner mitochondrial membrane. Moreover, another possibility of eliminating this compound is diffusion at the cellular level, where it is neutralized and removed with the aid of several antioxidant enzymes such as catalase, glutathione peroxidase, and thioredoxin peroxidase [23,49,50]. When it remains unmetabolized, H_2_O_2_ interacts with O_2_^•−^ and generates the hydroxyl radical (OH^•^), a molecule that is extremely reactive and destructive at the cellular level. Furthermore, in the mitochondrial outer membrane, there is a monoamine oxidase (MAO) enzyme that acts as another source of H_2_O_2_ [51], which explains the development of efficient H_2_O_2_ elimination mechanisms using these organelles [52]. Another radical entity is represented by the hydroperoxyl radical (HOO^•^), the protonated form of O_2_^•−^, which, however, physiologically develops in small amounts at the cellular level [53]. The latter may be involved in lipid peroxidation too [46]. Another enzyme located in the intermembrane space, p66Shc, has been identified as playing a role in ROS production [46,54]. Moreover, singlet oxygen (^1^O_2_) has been identified as playing an important role in the destruction of mtDNA [55]. ^1^O_2_ is a mitochondrial permeability modulator which can be generated through cytochrome c-catalyzed peroxidation, with carbonyl groups as the substrate [56].

Mitochondria are also a source of reactive nitrogen species (RNS). Nitric oxygen (NO^•^) is produced enzymatically by means of nitric oxide synthases (NOS) from amino acids [57]. L-arginine is metabolized in the presence of NOS, forming L-citrulline and NO^•^. NADPH and oxygen are also involved in this reaction [58]. Cytochrome c can act as an antioxidant promoting NO^•^ catabolism, but also O_2_^•−^ to O_2_ oxidation [59]. Another antioxidant system is the NAD(P)+-dependent transhydrogenase, located at the inner mitochondrial membrane. It maintains the amount of NADPH in the reduced form by catalyzing the transfer between NADH and NADP^+^. In addition, mitochondria contain alpha-tocopherol (vitamin E) and UQH2, inhibitors of lipid peroxidation [59] (Table 1).

Essential metals such as copper (Cu), manganese (Mn), zinc (Zn), and iron (Fe) are nutrients in various processes that take place at the intracellular level. Copper (Cu) is a cofactor for enzyme function and has an increased redox potential, allowing the transfer of electrons to oxygen and ROS production. An inadequate amount at the cellular level is associated with a compromised immune system, organ dysfunction, and oxidative damage [60]. Mn is a cofactor for essential enzymes, including catalase and Mn superoxide dismutase (Mn-SOD). Mn-SOD catalyzes O_2_^•−^ to H_2_O_2_ via the Mn^2+^/Mn^3+^ cycle, detoxifies free radicals, and prevents oxidative stress. Catalase converts H_2_O_2_ to oxygen and water, consequently reducing the production of oxidative stress. Due to these metalloproteins, Mn is involved in antioxidant defence, immune response, and energy production. Excess Mn has been correlated with oxidative stress and mitochondrial dysfunction. Mn can interfere at the mitochondrial level with oxidative phosphorylation, inhibiting F1-ATPase function and ATP synthesis. Oxidative stress caused by the pro-oxidant capacity of Mn results in increased mitochondrial solubility for protons and ions, a loss of mitochondrial membrane potential, changes in oxidative phosphorylation, and mitochondrial swelling. After chronic exposure to Mn, its accumulation at the mitochondrial level has been observed in neurons and astrocytes [61,62,63]. Increased amounts of Zn suppress the Cu and Fe absorption, causing the production of increased amounts of ROS at the mitochondrial level, disrupting the activity of various enzymes, and activating apoptotic processes. The imbalance of these metals causes structural and functional changes in enzymes, receptors, and transporters [63]. Intracellular Fe is found in its reduced form, and it is a cofactor for enzymes located in the cytosol, mitochondria, and nucleus. Catalase, one of the most important antioxidant enzymes, contains four haem groups. Free Fe can exchange electrons with surrounding molecules and form free radicals. Free Fe donates an electron to H_2_O_2_ and forms OH^•^ via the Fenton reaction [64].

Additionally, mitochondria can generate heat due to proton leak. The proton leak results from the activity of fatty acids on uncoupling proteins (UCPs). UCPs belong to the family of mitochondrial anion carrier proteins. Five UCPs have been identified in mammals (UCP1, UCP2, UCP3, UCP4, and UCP5) [65], and they have a purine nucleotide-binding site. ATP, ADP, GTP, and GDP are inhibitors of proton flux and ROS, and fatty acids are activators. UCPs can regulate ion transportation, calcium homeostasis, or synaptic plasticity. UCP1 is expressed in brown adipose tissue, and it is important in the maintenance of body temperature. UCP2-5 have different physiological actions in specific tissues, reducing oxidative stress. UCP2 is associated with metabolic disorders such as diabetes, obesity, and cardiovascular disease [13].

**Table 1:** ROS/RNS and promoters of free radicals; antioxidant systems; and positive and negative effects of oxidative stress on diabetes, obesity, and cardiovascular disease.

**ROS/RNS and Promoters of Free Radicals**	**Antioxidants System**	**Positive Impacts of Free Radicals**	**Negative Impacts of Free Radicals**	**Ref.**
Superoxide radical anion (O_2_^•−^) Hydrogen peroxide (H_2_O_2_) Monoamine oxidase (MAO) Singlet oxygen(^1^O_2_) Hydroxyl radical (OH^•^) Hydroperoxyl radical (HOO^•^) Nitric oxide (NO^•^)	Superoxide dismutase (SOD): Mn-SOD, Cu/Zn-SOD Catalase Glutathione peroxidase Thioredoxin peroxidase NAD/NADP transhydrogenase Cytochrome c oxidase Vitamin E UQH2	Signalling pathways and cell structures synthesis (within fibroblasts, endothelial cells, vascular smooth muscle cells, cardiac myocytes) Immune system activity rise Induction of mitogenic response Vasodilation Angiogenesis Wound healing	Lipid peroxidation Damage to cell membranes and lipoproteins Cytotoxic and mutagenic compounds Conformational modifications of proteins DNA lesions Loss of epigenetic information Hypertension Atherosclerosis	[66,67]
**Disease**	**Biomarkers**	**Mechanism of action and effects of oxidative stress**		
Diabetes	↑malondialdehyde ↑8-isoprostane ↑4-hydroxynonenal ↑glycated haemoglobin ↑advanced oxidation protein products ↑protein carbonyls ↓glutathione ↓superoxide dismutase ↓catalase	Lipid peroxidation Protein oxidation Decreased insulin activity Hyperglycaemia Stimulation of the polyol pathway Stimulation of glucose autoxidation Increase in advanced glycosylation end products mtDNA and proteins conformational modifications		[23,68,68,69,70,71]
Obesity	↑tumour necrosis factor-α ↑nuclear factor-κB ↑interleukin-1β ↑interleukin 6 ↑plasminogen activator inhibitor 1 ↓superoxide dismutase ↓catalase ↓vitamin A ↓vitamin E ↓vitamin C	Excess of pro-inflammatory cytokines and expression of adhesion molecules and growth factors Depleted antioxidant levels Increase in free fatty acids Thrombosis and insulin resistance		[72]
Cardiovascular Disease	↑oxidized low-density lipoprotein ↑tumour necrosis factor-α ↑nuclear factor-κB ↑interleukin-1β ↑interleukin 6 ↑8-Hydroxyl-2′-deoxyguanosine ↑myeloperoxidase ↑F2-isoprostanes ↑biopyrrins ↓vitamin C ↓glutathione peroxidase 1 ↓total antioxidant status	Endothelial dysfunction Inflammation in blood vessels Atherosclerosis Hypertension Cardiac hypertrophy Cardiomyocytes apoptosis Oxidative damage in DNA Lipid peroxidation		[73,74,75,76,77,78,79,80]

Note: ↑ is increase, ↓ is decrease.

## 4. Insulin Resistance, Diabetes and Mitochondrial Dysfunctions

Type 2 diabetes mellitus (T2DM) is a chronic pathology that requires continuous medical care through pharmacological treatment, but also a reduction in risk factors involved in the etiopathogenesis of the disease. Currently, this condition is characterized by a permanent increase in incidence and prevalence [81]. In recent years, we notice an increase in interest towards research and the identification of mitochondrial changes and their involvement in chronic diseases. There are numerous studies which confirm that excess ROS and the presence of mitochondrial dysfunctions contribute to the development of metabolic pathologies and insulin resistance. Obesity, diabetes, and cardiovascular disease have been linked to mitochondrial dysfunction [82,83,84,85,86].

Hyperglycaemia and T2DM are directly linked to mitochondrial activity, function, and oxidative stress. Mitochondria produce the largest amount of ROS and ATP. With regards to hyperglycaemic status, the amount of ROS increases and triggers changes in cellular homeostasis with the generation of lesions at this level [87]. The increased production of O_2_^•−^ affects the translocation capacity of glucose transporter 4 (GLUT4) from the intracellular level to the plasma membrane, resulting in a decrease in insulin action at the tissue level and an increase in blood glucose amount [70,71]. Hyperglycaemia generates an excessive production of ROS which favours the appearance of mitochondrial changes and stimulates the polyol pathway, glucose autoxidation, and an increase in advanced glycosylation end products in diabetic patients [23,88]. Changes at the OXPHOS level, a reduction in NADH oxidoreductase, and citrate synthase activity induce insulin resistance [89]. Fatty acid catabolism, a mechanism called beta-oxidation, is also carried out at the mitochondrial level. The reduction in fatty acid oxidation, together with the accumulation of lipids and diacylglycerol, drive both the activation of protein kinase C and increased ROS production. Thus, there are changes in the mitochondrial functioning mechanism that affect ATP synthesis. A compromise of mitochondrial function causes a lipid excess and the development of insulin resistance [89,90]. The amplification of oxidative stress generates and maintains inflammation, causes lipid peroxidation, and initiates changes in the insulin signalling mechanism. Insulin resistance generated by hyperlipidaemia induces changes in mtDNA and proteins [70].

**Figure 2:**
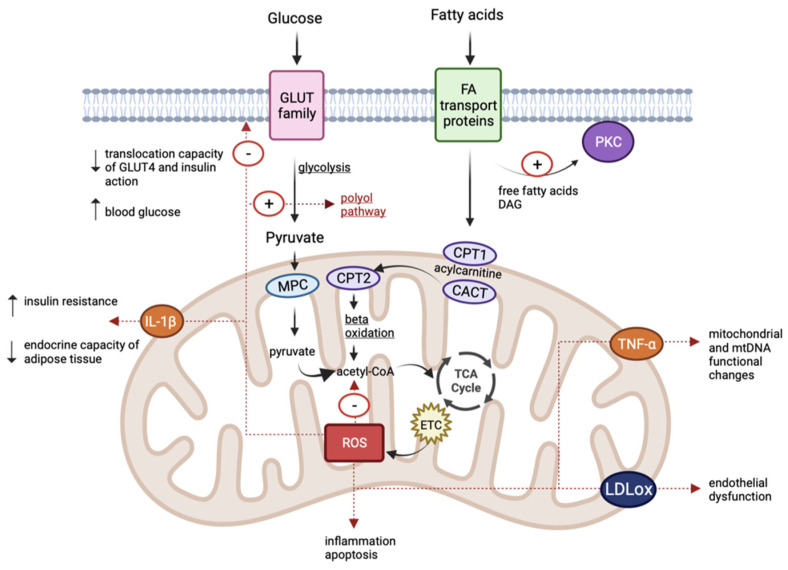
Schematic representation of common pathophysiological mechanisms in diabetes, obesity, and cardiovascular disease. (TCA, tricarboxylic acid cycle; ETC, electron transport chain; CPT1, carnitine palmitoyl-transferase 1; CPT2, carnitine palmitoyl-transferase 2; CACT, carnitine-acylcarnitine translocase; MPC, mitochondrial pyruvate carrier; IL-1β, interleukin IL-1β; TNF-α, tumour necrosis factor-α; LDLox, oxidized LDL; DAG, diacylglycerol).

In diabetic patients, at the level of mononuclear cells, increased amounts of ROS were identified, as well as spherical and hyperpolarized mitochondria, thus indicating dysfunctions at this level [91]. The effect of ROS was also proven in pancreatic β cells; the changes including volume and shape modifications, as well as changes in mitochondrial function. They affect ATP-dependent K+ channels and insulin secretion. This aspect can also be explained by the lower amount of antioxidants in β-cells [92]. The reduction in ATP production and the increase in ROS at the muscle level can trigger an increase in insulin resistance and diabetes [93] (Figure 2).

Changes in mtDNA and genomic DNA have also been identified in patients with diabetes mellitus. A new *m.8561C>G* mutation in *MT-ATP6/8* was identified and correlated with T2DM and hypergonadotropic hypogonadism [94]. The *m.3242A>G* mutation and the *10.4-kb deletion* were associated with diabetes and deafness. It is considered that mitochondrial mutations can accumulate over time (an aspect that correlates with the aging process and neurodegeneration) [86]. There is also a correlation between mitochondrial epigenetic changes and insulin resistance [95]. These changes at the mtDNA level suggest the importance of continuing research in this field. Changes in the *COX7A1* and *NDUFB6* genes have been identified in people with insulin resistance and T2DM. Although they are nuclear genes, they encode subunits of OXPHOS complexes I and IV, respectively [82,95,96].

## 5. Obesity and Mitochondrial Dysfunctions

Obesity is associated with changes in carbohydrate and lipid metabolism, but also with insulin resistance, an increased risk of cardiovascular pathologies, and diabetes mellitus [97]. It represents one of the components of the metabolic syndrome and a cause of the development of numerous chronic conditions. A caloric imbalance causes the hyperplasia and hypertrophy of adipose cells. Adipose tissue secretes adipokines that have immunoregulatory properties [98]. The maintenance of the inflammatory process by an increase in leptin and resistin (pro-inflammatory factors) and decrease in adiponectin (an anti-inflammatory factor) triggers an increase in ROS and oxidative stress [98,99]. Mitochondrial dysfunctions cause interleukin IL-1β secretion, which affects peripheral insulin sensitivity and interferes with the endocrine capacity of the adipose tissue [100]. In hyperlipidaemic diets, mitochondrial fatty acid oxidation increases, inducing the subsequent generation of increased levels of acetyl coenzyme A, which further amplifies the levels of NADH and FADH2 in the tricarboxylic acid cycle, as well as the accumulation of acylcarnitine and ROS formation. The excess of free fatty acids at the adipocyte level activates NADPH oxidase enzymes with an increase in ROS. Oxidative stress causes inflammation and boosts lipid peroxidation, disrupting insulin’s mechanisms of action [70,99,101] (Figure 2).

Cells can release extracellular vesicles containing mitochondria. Recipient cells receive mitochondria through an extracellular vesicle–cell fusion event [102]. The intercellular transfer of mitochondria has been implicated in many pathological conditions such as stroke, pulmonary hypertension, and obesity [103].

Adipocytes transfer mitochondria to macrophages in adipose tissue, generating a new population of macrophages, which is greatly diminished in patients with obesity due to increased lipid intake because of reduced mitochondrial uptake by macrophages. Mitochondrial uptake is mediated by heparan sulphate and it manifests low levels in obese subjects. The exostosin (EXT) 1 gene and the EXT2 heterodimer are also associated with the maintenance of lipid metabolism homeostasis and glucose levels [95]. The presence of deletions in the EXT1 gene in myeloid cells reduces heparan sulphate levels, decreases mitochondrial transfer, and increases adipose tissue accumulation [104].

It has been shown that numerous components of the ETC have decreased expression in visceral adipose tissue in women with diabetes. A decrease in the expression of OXPHOS genes was also noted in these patients [105]. A reduction in the number of mtDNA copies at blood, muscle, and adipose tissue level has been reported in obese subjects and those with type 2 diabetes mellitus [106,107]. An association between the reduction in mtDNA copy numbers and increased mtDNA methylation in the D-loop region was identified in obese individuals [95].

## 6. Cardiovascular Disease and Mitochondrial Dysfunctions

Cardiovascular disease is the leading cause of death worldwide. There are a large number of factors involved in the development of cardiovascular pathologies. Mitochondrial changes produced during the aging process explain the functional deficit encountered in cardiovascular pathologies [108].

Cardiomyocytes contain numerous mitochondria to generate a large amount of ATP [109]. At the cardiac level, mitochondria are located in interfibrillar, subsarcolemmal, and perinuclear regions [110]. Mitochondrial structural changes occur in cardiac pathologies, with the formation of megamitochondria (giant mitochondria generated by fusion). Shape changes are also encountered (cristae reorientation and the presence of intramitochondrial cylinders) [80].

The largest amount of ATP is obtained at the cardiac level via the beta-oxidation of fatty acids but, depending on availability, glucose can be used as an energy source. In cardiac pathologies, insulin signalling is altered, affecting metabolic flexibility. Therefore, the amount of ATP decreases [80].

Oxidative stress and mtDNA changes are identified in patients with cardiovascular pathologies. At the cardiac level, ROS are generated through complexes I and III in neutrophils, endothelial cells, and myocytes. The increase in ROS causes endothelial dysfunction, inflammation in blood vessels, and the generation of oxidized LDL at the arterial level. Ultimately, these changes trigger atherosclerosis, hypertension, and cardiac hypertrophy. In addition, mitochondrial dysfunctions stimulate enzyme activation and induce cardiomyocyte apoptosis [79,80]. It has been found that ROS association, together with the increase in pro-inflammatory factors such as tumour necrosis factor-α (TNF-α), causes mitochondrial and mtDNA functional changes, favouring the development and progression of cardiovascular pathologies [111] (Figure 2).

Several studies indicate methylation changes in cardiovascular pathologies. Extensive methylations were found in the MT-CO1, MT-CO2, MT-CO3, and MT-TL1 genes in subjects with these conditions, with the methylation degree of these genes being a potential predictive marker of cardiovascular pathologies in obese patients [112].

## 7. Pharmacological Strategies and Lifestyle Interventions in Mitochondrial Dysfunctions

Mitochondrial dysfunctions are involved in the pathophysiological mechanisms that generate metabolic disorders. In this context, interest shown toward mitochondria can generate new optimal therapies for these pathologies. The aim is to slow the progression of mitochondrial dysfunctions and decrease ROS with a subsequent reduction in oxidative stress.

Recent studies support the idea that physical activity improves insulin sensitivity and mitochondrial function in muscle tissue in patients with T2DM. Caloric restriction reduces excessive ROS production and inhibits inflammation, thus facilitating the prevention and treatment of metabolic pathologies [113,114,115]. In recent years, it has been suggested that a diet rich in polyphenols might be beneficial in subjects affected by metabolic dysfunction [116].

Due to the multitude of discoveries in the field, interest in mitochondrion-directed therapy has increased. Although numerous action mechanisms are known, the challenge is represented by the transportation of active substances at this level due to the barriers that precede mitochondrial localization. In this regard, some of the drugs that may represent a new therapeutic solution in metabolic pathologies are selected further.

ETC components may represent a target in pharmaceutical interventions. Rotenone and *Annonaceous acetogenins* inhibit NADH ubiquinone oxidoreductase. Moreover, metformin is a complex I inhibitor, a drug used in diabetes that increases glucose consumption. Vitamin E analogues (α-tocopheryl succinate) and 3-bromopyruvate act on complex II. Complex III inhibitors are antimycin A and myxothiazole. Cyanide is a complex IV inhibitor. There are numerous pharmaceutical agents that act on complex V (oligomycin, apoptolidins, resveratrol, dindolyl methane, and aurovertin) [117,118].

A subfamily of mitochondrial proteins is represented by uncoupling proteins (UCPs). UCPs facilitate H+ transport, ultimately generating heat. Fatty acids stimulate UCP transport. In addition, it was highlighted that adrenaline and prostaglandins determine the upregulation of UCPs. They are involved in common pathologies, with UCP1 and UCP2 being associated with diabetes and obesity. Carbonyl cyanide m-chlorophenyl hydrazone and 2,4-dinitrophenol are uncoupling agents for oxidative phosphorylation. UCPs represent a good pharmacological target for treating obesity and diabetes [118,119,120].

From a pharmacological point of view, sirtuins (silent information regulator proteins) are involved in the regulation of glucose and lipid metabolism at the cellular level [121,122,123]. Resveratrol activates SIRT 1, and improves insulin resistance through its antioxidant properties [124].

Mitochondrial fission inhibitors such as mitochondrial division inhibitor 1 (Mdivi 1), P110, and dynasore have been identified as playing a role in ameliorating oxidative stress [125,126,127]. Mdivi 1 inhibits the GTPase activity of DRP1, improves myocardial infarction after ischemia, and restores mitochondrial changes caused by excess ROS. It was demonstrated in a study conducted on an animal model that Mdivi 1 reduces inflammation and oxidative stress and improves endothelial function [94]. Dynasore inhibits the mitochondrial fission phenomenon and increases the survival chances of cardiomyocytes after ischemia. P110 is a peptide that inhibits DRP1 activity, with a beneficial effect on mitochondrial morphology and function. Its effect is demonstrated in cancer and neurological pathologies, but in metabolic dysfunctions, it is still being studied [94]. Cyclophilin D is a mitochondrial protein known to regulate the mitochondrial permeability transition pore [128]. This can represent a therapeutic target.

## 8. Antioxidants

Several studies have highlighted the beneficial effect of antioxidant use in metabolic pathologies [129,130,131,132]. An increase in antioxidants, such as vitamin E, vitamin C, coenzyme Q, and N-acetylcysteine (NAC), is indicated for their oxidative stress reduction [133,134]. NAC is the acetylated form of the amino acid L-cysteine and is a source of the thiol group (SH) with the potential to stimulate glutathione biosynthesis. It inhibits the activity of p38 MAP kinase, nuclear factor kappa B, and redox-sensitive activating protein-1; increases SH availability; and interacts with NO^•^ forming nitrosothiols. NAC inhibits the oxidative degradation of NO^•^, thus decreasing the amount of nitrogen dioxide, peroxynitrite, and nitrotyrosine. NAC therapy has beneficial effects on oxidative stress, inflammation, epithelial dysfunction, and hypertension [135]. Coenzyme Q10 (CoQ10) is an antioxidant and plays an important role in lipid structures (cell membranes and lipoproteins). It is localized at the mitochondrial and extramitochondrial levels and has three oxidation states (oxidized, partially reduced, and fully reduced). Combining oxidized CoQ10 with selenium improves the protection against cardiovascular pathologies. Patients with T2DM have low amounts of COQ10, and supplementation with CoQ10 and selenium leads to the reduced formation of advanced glycosylation end products. Moreover, the use of CoQ10, together with selenium and vitamin C, has synergistic antioxidant effects [136].

Although the beneficial effect of using conventional antioxidants in metabolic pathologies is known, in some clinical trials, the results of their benefits are contradictory. Salehi et al. highlighted the adverse effects of inappropriately using nontargeted antioxidants (vitamin A, vitamin C, vitamin E, and β-carotene). Excessive vitamin A intake (more than 10,000 IU) has been associated with increased teratogenicity risk or birth defects. Vitamin C can be metabolized to oxalate, increasing the risk of calcium oxalate kidney stones, and vitamin E may increase prostate cancer risk [137]. The excessive use of antioxidants can decrease ROS production and stimulate the compensatory upregulation of mitogen-activated protein kinase (MAPK) pathways. Furthermore, it is difficult to calculate the dose of nontargeted antioxidants used for disease treatment because there are many uncertainties about the mechanism of absorption and how they are metabolized in different organs. Due to the adverse effects of conventional antioxidants at the cellular level, the production and use of mitochondria-targeted antioxidants are becoming more relevant [137,138].

Mitoquinone (MitoQ), mitovitamin E (MitoE), and MitoTEMPO are mitochondria-targeted antioxidants and are synthesized by attaching the antioxidant to the lipophilic triphenylphosphonium cation (TPP+). TPP+ is the most widely used lipophilic cation. To permeate the mitochondrial membrane, the substances must permit the optimal lipophilic level. If the molecule has low lipophilicity, it does not penetrate the mitochondrial membrane, and if lipophilicity is increased, it accumulates at the membrane level [109]. Due to the processes carried out in the ETC, the increase in the mitochondrial membrane potential allows the passage of these modified antioxidants inside the mitochondria and the action at this level. MitoQ is an antioxidant in which TPP+ is bound to the UQ. It accumulates at the mitochondrial membrane, decreases the production of ROS, and prevents the potential changes caused by oxidative stress [139,140]. MitoQ is now described as a potential pharmaceutical compound in neurodegenerative pathologies [141,142,143]. MitoE is a therapeutic agent that crosses the mitochondrial membrane and protects mitochondria against excess ROS [144]. SkQ1 is also a mitochondrial antioxidant formed from the conjugation of TPP+ with plastoquinone [145]. SkQ1 has antioxidant effects at low concentrations and can bind to cardiolipin, preventing its oxidation. MitoQ and SkQ1 can reduce oxidative stress, decrease protein oxidation, and prevent lipid peroxidation and cell apoptosis. Animal model studies have revealed their benefits in metabolic syndrome, obesity, and ischemia–reperfusion injury [109]. Mitochondrial toxicity is the limiting factor in the use of TPP+ antioxidants. This requires proper dosing and concentrations below levels at which mitochondrial membrane damage occurs [138].

Liposomes are bilipid membrane vesicles used to transport certain bioactive substances. Liposome-encapsulated antioxidants are composed of cholesterol, phosphatidylcholine, phosphatidylglycerol, cholesterol, and antioxidants (quercetin, NAC, and vitamin E). Liposome-encapsulated antioxidants penetrate at the cellular level through the phenomenon of pinocytosis, the liposomal components fuse with the mitochondrial membrane, and the antioxidant is released at the mitochondrial level [138]. MITO-Porter is a novel system used for transporting bioactive components to the mitochondrial level.

It represents a liposomal nanocarrier made up of 1,2-dioleoyl-sn-glycerol-3-phosphatidylethanolamine, sphingomyelin, and stearylated octa arginine peptide (R8). MITO-Porter binds at the mitochondrial level due to the interaction between R8 and negatively charged mitochondria [146].

## 9. Conclusions and Perspectives

Mitochondria are versatile organelles, responsible for most of the cellular chemical energy production. The structure and function of this subcellular organelle are peculiar, due to the abounding molecular mechanisms that take place at this level. They are responsible for cell survival, apoptosis, and homeostasis. These benefits coexist with the high degree of errors that can occur at the mitochondrial level through ROS. Mitochondrial changes have been identified in metabolic dysfunctions including insulin resistance/diabetes, hypertension, and dyslipidaemia, but also in cancer or neurodegenerative pathologies. Recent studies have identified changes in mitochondrial biogenesis and dynamics, as well as the presence of oxidative stress in metabolic dysfunctions.

In diabetic patients, at the level of mononuclear cells, spherical and hyperpolarized mitochondria were identified, indicating dysfunctions at this level. In pancreatic β cells, changes including volume, shape modifications, and mitochondrial dysfunction have been reported as well. The reduction in ATP production and the increase in ROS at the muscle level can trigger an increase in insulin resistance and diabetes. In obesity patients, the excess of free fatty acids at the adipocyte level activates NADPH oxidase enzymes, with an increase in ROS production. Oxidative stress causes inflammation and boosts lipid peroxidation, disrupting insulin’s mechanisms of action. The increase in ROS causes endothelial dysfunction, inflammation in blood vessels, and the generation of oxidized LDL at the arterial level in cardiovascular diseases.

To reduce the progression of metabolic pathologies, lifestyle interventions, physical exercises, and dietary changes are indicated. A balanced diet, including fruits, legumes, and vegetables of different colours, is recommended. Moreover, new pharmaceutical strategies that can improve the prognosis of these conditions are being investigated and developed. Several studies have highlighted the beneficial effect of using antioxidants in metabolic pathologies; vitamin E, vitamin C, coenzyme Q, and NAC administration are indicated for their oxidative stress reduction effect. However, in some clinical trials, the results of their benefits are contradictory. Due to the adverse effects of conventional antioxidants at the cellular level, the production and use of mitochondria-targeted antioxidants are becoming more relevant.

The epigenetic role in this context is not fully understood, but changes in mtDNA have been identified which could be meaningful in the identification and design of the next therapeutic strategies. It is considered that mitochondrial mutations can accumulate over time. Several studies indicate methylation changes in cardiovascular pathologies, and the methylation degree of specific genes can be a potential predictive marker of cardiovascular pathologies in obese patients. Difficulties in this field arise due to the particularities encountered at the level of each individual. Large and complex studies are needed in order to identify and detail the changes at the mitochondrial level and the therapeutic approaches.

## Abbreviations

ATP	adenosine triphosphate
OXPHOS	oxidative phosphorylation
mtDNA	mitochondrial DNA
ROS	reactive oxygen species
MS	metabolic syndrome
T2DM	type II diabetes mellitus
NADH	reduced nicotinamide adenine dinucleotide
FADH2	reduced flavin adenine dinucleotide
TCA	tricarboxylic acid
ETC	electron transport chain
UQ	ubiquinone
UQH2	ubiquinol
Q-	semi-quinone radical ion Q-
Hs	heavy strand
Ls	light strand
NCR	non-coding region
D-loop	displacement loop
OH	origin of heavy-strand synthesis
HSP	heavy-strand promoter
LSP	light-strand promoter
TLR9	toll-like receptor 9
TOM	translocase of the outer mitochondrial membrane
TIM	translocase of the inner mitochondrial membrane
Mfn	mitofusins
OPA1	optic atrophy 1 protein
Drp1	dynamin-related protein 1
Fis1	fission protein
PGC-1α	proliferator-activated receptor gamma coactivator-1α
NRF	nuclear respiratory factor
TFAmt	mitochondrial transcription factor
AMPK	AMP-activated protein kinase
SIRT1	silent information regulator 1
O_2_^•−^	superoxide radical anions
H_2_O_2_	hydrogen peroxide
MAO	monoamine oxidase
^1^O_2_	singlet oxygen
OH^•^	hydroxyl radical
HOO^•^	hydroperoxyl radical
NO^•^	nitric oxide
RNS	reactive nitrogen species
NOS	nitric oxide synthases
GLUT4	glucose transporter 4
EXT	exostosin
NAC	N-acetylcysteine
COQ10	coenzyme Q10
MitoQ	mitoquinone
MitoE	mitovitamin E
TPP+	triphenylphosphonium cation
